# FTIR nanobiosensors for *Escherichia coli* detection

**DOI:** 10.3762/bjnano.3.55

**Published:** 2012-07-03

**Authors:** Stefania Mura, Gianfranco Greppi, Maria Laura Marongiu, Pier Paolo Roggero, Sandeep P Ravindranath, Lisa J Mauer, Nicoletta Schibeci, Francesco Perria, Massimo Piccinini, Plinio Innocenzi, Joseph Irudayaraj

**Affiliations:** 1 Nucleo Ricerca Desertificazione, Università degli Studi di Sassari, Viale Italia 57, 07100 Sassari, Italy; 2 Department of Agricultural and Biological Engineering and Bindley Bioscience Center, Purdue University, 225 S. University Street, West Lafayette, 47907, Indiana; 3 Lea Nanotech s.r.l. S.P. 55 Porto Conte/Capo Caccia, km 8.400 località Tramariglio, 07041 Alghero (SS), Italy; 4 Dipartimento di scienze zootecniche, Università degli Studi di Sassari, Via Enrico De Nicola 9, 07100 Sassari, Italy; 5 Department of Food Science, Purdue University, 745 Agriculture Mall Drive, West Lafayette, 47907, Indiana; 6 Biodiversity s.r.l. S.P. 55 Porto Conte/Capo Caccia, km 8.400 località Tramariglio, 07041 Alghero (SS), Italy; 7 Porto conte ricerche, SP 55 Porto Conte/Capo Caccia, km 8.400 località Tramariglio, 07041 Alghero (SS), Italy; 8 Materials Science and Nanotechnology Laboratory, D.A.P., CR-INSTM, Università di Sassari, Palazzo Pou Salit, Piazza Duomo 6, 07041 Alghero (SS), Italy

**Keywords:** biosensors, *E. coli*, FTIR spectroscopy, foodborne pathogens, nanomaterials

## Abstract

Infections due to enterohaemorrhagic *E. coli* (*Escherichia coli*) have a low incidence but can have severe and sometimes fatal health consequences, and thus represent some of the most serious diseases due to the contamination of water and food. New, fast and simple devices that monitor these pathogens are necessary to improve the safety of our food supply chain. In this work we report on mesoporous titania thin-film substrates as sensors to detect *E. coli* O157:H7. Titania films treated with APTES ((3-aminopropyl)triethoxysilane) and GA (glutaraldehyde) were functionalized with specific antibodies and the absorption properties monitored. The film-based biosensors showed a detection limit for *E. coli* of 1 × 10^2^ CFU/mL, constituting a simple and selective method for the effective screening of water samples.

## Introduction

Foodborne illness is primarily caused by pathogenic microorganisms among which *Campylobacter, Salmonella, Listeria monocytogenes*, and *Escherichia coli* O157: H7 are responsible for the majority of foodborne outbreaks [1–2], and most of the recalls of food products have implicated these pathogens [3]. In particular *E. coli* is a typical inhabitant of the human intestinal tract; however, the strain *E. coli* O157:H7, one of the most dangerous foodborne pathogens [4], causes diseases that may lead to death [5]. Sensors to detect these pathogens, especially in ground beef and raw milk [6], are critical.

The conventional methods for the detection and identification of pathogens [7–8] are mostly based on (i) culture and colony counting methods (which involve counting of bacteria) [9]; (ii) immunology-based methods (which involve antigen–antibody interactions) [10]; and (iii) the polymerase chain reaction (PCR) method (which involves DNA analysis) [11–12]. While these methods are sensitive and can recognize pathogens, the type of organism and the number of colonies in a qualitative and quantitative manner, they are not efficient because of the enrichment step necessary to detect pathogens in low numbers, to comply with the safety regulations of the food and water supply chain. In addition, these methods are expensive, complicated, time-consuming, and require skilled personnel with expertise in molecular biology.

To overcome these problems, devices that can be used in situ, and that are simple, highly sensitive, inexpensive and rapid are attractive because they can be used for rapid screening of different samples for timely detection of these pathogenic agents. In fact monitoring food quality and safety attributes, by using new detection methods that have the potential to be sensitive and rapid [13], is important to prevent and identify problems related to health and safety. Lately, micro- and nanosystems for bacteria and food sample analysis have been developed as innovative tools for the detection of foodborne pathogens in the food and drink industries [14–21]. In particular, different optical biosensors were created for rapid detection of pathogenic bacteria, using fluorescence or surface plasmon resonance (SPR) because of their sensitivity [22–23].

For fluorescence analysis, antibodies (Ab) are conjugated with fluorescent compounds and used in combination with classical techniques (ELISA, PCR). With these biosensors overnight culture is required, leading to a long time for the analysis, while SPR is a powerful and complex technique, which unfortunately requires specialized staff, and costly and large equipment [24–25]. To overcome these limitations the aim of the present work is to develop a simple new nanodevice capable of detecting pathogens in low concentration and suitable for a fast real-time monitoring, using Fourier transform infrared (FTIR) spectroscopy as an optical transduction method.

## Experimental

### Chemicals

All commercially available solvents and reagents were used without further purification. Titanium tetrachloride (TiCl_4,_ >98%), anhydrous ethanol (EtOH, >99.9%), bidistilled water, acetone (>99.8%), and toluene (>99.5%), were purchased from Carlo Erba (Italy). Pluronic F-127 (cell culture test), (3-aminopropyl)triethoxysilane (APTES, >98%), glutaraldehyde (GA Grade I, 50% in H_2_O, specially purified for use as an electron microscopy fixative or other sophisticated use) were purchased from Sigma Aldrich (Germany). *E. coli* O157:H7 and *E. coli* K12 were obtained from the bacteria collection at Purdue University (Agricultural and Biological Engineering). BHI agar, PCA, and LB were purchased from Teknova (Hollister, CA). Bac-trace affinity purified antibodies goat anti-*E. coli* O157:H7 were purchased from Kirkegaard and Perry Laboratories Inc. (Gaithersburg, MD). Silicon wafers (test grade, p-type boron doped, diameter 4″, thickness 475–575 μm, (100) oriented (one side polished and one side etched) were obtained from Jocam (Italy).

### Film preparation

Titania (TiO_2_) thin films were prepared by dipping silicon wafers in a solution composed of TiCl_4_/Pluronic F127/H_2_O/EtOH (1:0.005:10:40) under controlled conditions of temperature and RH (relative humidity). Films were deposited with a dip coater, aged at room temperature (RH 50% for 24 h) and, to increase the inorganic polycondensation and stabilize the mesophase, the films were submitted to different firing steps at 60, 120 and 200 °C for 24 h at each temperature in an oven at a heating rate of 10 °C·min^−1^. The final calcination process to remove the organic template of these stabilized coatings was conducted at 350 °C for 3.5 h in air under static conditions at a heating rate of 10 °C·min^−1^. In this way mesoporous titania thin films were obtained and characterized as described in a previous work of our group [26].

### Film functionalization

The optimization of the functionalization with amino-groups was obtained by immersing the calcined films in a solution 0.2 M of APTES in toluene for 24 h at 25 °C. The amino grafted films were carefully washed with toluene over several washing cycles and finally dried in air. The following functionalization of TiO_2_–APTES films with GA was obtained by immersing the films in GA 50% (v/v) in water for 24 h, washing with water and EtOH and drying at room temperature. Different experiments were realized, providing the immobilization of antibodies directly on titania thin films, on films functionalized with APTES, or on films functionalized with APTES and GA. Antibody solutions were prepared by dissolving 200 μL of anti-*E. coli* O157:H7 in 800 µL phosphate buffered saline (PBS) to achieve a final concentration of 50 μg/mL, after which the films were covered with this solution for 15 h at 4 °C. Finally the films were washed with PBS and water, and dried at room temperature. FTIR spectroscopy measurements were carried out for each step to monitor the chemical functionalization and the linking of antibodies to the films.

### Bacteria preparation

*E. coli* O157:H7 and *E. coli* K12 were cultured on agar plates for 24 h then a single colony of each species was transferred into 10 culture tubes containing 5 mL each of Luria-Bertani medium (LB) and placed in an incubator at 37 °C, under shaking, for 18 h, at 120 rpm. Then the tubes were centrifuged at 3500 rpm for 10 min, to obtain a pellet. Finally LB was removed from the tube and the cells were washed three times with sterile PBS to remove residual medium and resuspended in 3 mL PBS for binding experiments.

Serial dilutions of bacteria were prepared for the detection step. To validate the data, a standard method for the counting of pathogens was also used: the culture and colony counting method. To revitalize the culture of *E. coli*, a single colony was transferred in a test tube containing a nutrient broth and placed in an incubator for 24 h at 37 °C. Then serial dilutions were carried out in sterile saline solution to a dilution of 10^−7^. Then 1 mL of solutions at a dilution of 10^−6^ and 10^−7^ were plated in a petri plate with plate count agar (PCA) by the inclusion method (in duplicate for each dilution). Finally, after cooling, the four plates were incubated at 37 °C for 24 h and the colonies were counted.

Considering the dilution factor and the mean number of colonies counted in two plates at a dilution of 10^−6^, the concentration of bacteria in the initial nutrient broth was obtained and estimated to be 1.13 × 10^8^ colony-forming units (CFU)/mL. For DNA analysis a standard method was used to identify the pathogen: real-time PCR. The instrumentation BioRad IQ5 was used for the analysis. After DNA extraction with the boiling method, PCR was carried out with a blank sample, a reference sample and the DNA extracted from 1 mL of nutrient broth; 50 cycles were programmed and an amplification at the 17th cycle could be observed.

### Determination of the detection limits of *E. coli* O157:H7

Functionalized titania thin films were incubated with freshly prepared anti-*E. coli* O157:H7 antibodies, and then different films were covered with 1 mL of *E. coli* O157:H7 suspension at concentrations ranging between 10^8^ and 10 CFU/mL for 30 min, to allow the binding to take place. Finally these films were washed and dipped for 15 min in PBS solution, washed with PBS and water, dried in air at room temperature and finally analysed by FTIR spectroscopy to determine the sensitivity of the method. Colony micrographs of these films were collected. Some tests with *E. coli* K12 were carried out to monitor the selectivity of the device.

### Film characterization

Mesoporous titania thin films were characterized with a Nicolet Nexus FTIR spectrophotometer equipped with a KBr–DTGS detector and a KBr beam splitter. The measurements were carried out in the range of 4000–700 cm^−1^ with 256 scans at 4 cm^−1^ resolution. The detector was cooled with liquid nitrogen for 60 min before data collection and also during the measurements.

The spectra of films deposited on silicon wafers were obtained in transmission mode. The background was recorded by using a silicon substrate. Atomic force microscopy (AFM) measurements were taken on titania films with an Asylum Research 3-D AFM in contact mode. An olympus BX-51 optical microscope with a 100× objective was used to collect microscopic images of films after pathogen immobilization.

## Results and Discussion

An optical biosensor was developed for the detection of pathogenic *E. coli* O157:H7, by using FTIR spectroscopy to provide mid-infrared fingerprints of pathogens present in buffer. The spectroscopic fingerprint of pathogens originates from the various functional groups related to proteins, lipids, and carbohydrates, and their mid-infrared (MIR) spectra can be used for the identification and structural characterization of different pathogens and subspecies [27]. MIR spectra are additive and sensitive, and allow the fingerprinting and quantification of the pathogen of interest, transforming the traditional devices into biosensing systems with high sensitivity.

**Figure 1:**
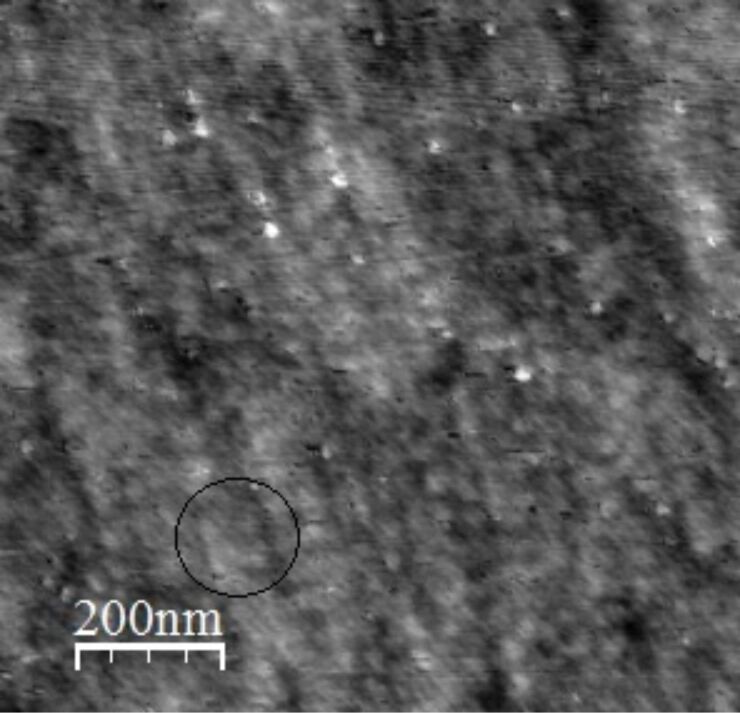
Microscopically ordered structure of a mesoporous titania film observed by AFM analysis.

In particular, mesoporous titania thin films synthesized with the sol–gel method, were used to immobilize biomolecules (antibodies and pathogens) thanks to the high surface area due to their nano-organization, visible in a AFM image (Figure 1). This was possible due to a high control of the gelation process on the films and subsequent thermal treatments that avoided the denaturation of biomolecules in environments that have a high alcohol concentration and extreme pH values, hence obtaining ordered and reproducible substrates. With this objective, special attention was paid to the thermal treatments of films to completely remove the inorganic template, EtOH and HCl.

Mesoporous titania was used as a substrate for the features described above, but also because it has excellent biocompatibility, stability (12 months at RT), and reproducibility, and can interact with biological molecules due to the formation of coordinated linkages between titania films, organic crosslinkers and amino or carboxyl groups of the antibodies or bacteria. In the present work the immobilization of bacteria was studied directly on titania films, on titania films functionalized with APTES and on titania films functionalized with APTES, GA and antibodies (Ab).

### Detection of *E. coli* with TiO_2_–APTES–GA–anti *E. coli* O157:H7-Ab

**Figure 2:**
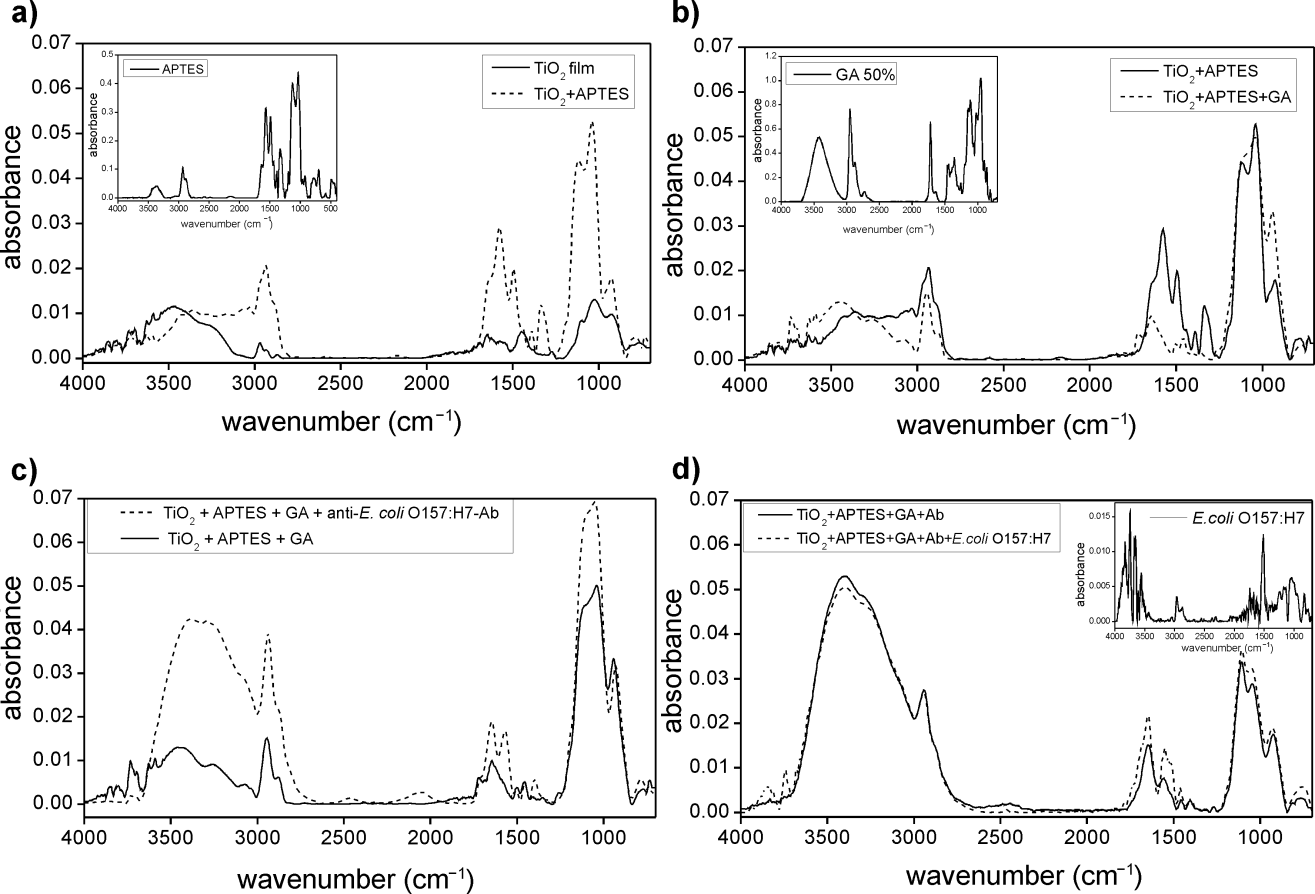
(a) FTIR spectrum of mesoporous titania thin films (solid line) and films functionalized with APTES (dashed line). The reference spectrum of APTES is reported in the inset. (b) FTIR spectrum of titania films functionalized with APTES (solid line) and after the linking of glutaraldehyde (dashed line); the spectrum of glutaraldehyde (GA) was reported in the inset for reference. (c) Spectrum of functionalized titania with APTES and GA (solid line) and after the linking of anti-*E. coli* O157:H7-antibody (dashed line). (d) FTIR spectrum of a titania film functionalized with APTES–GA–Ab (solid line) and after the immobilization of *E. coli* O157:H7 (dashed line); the reference spectrum of *E. coli* O157:H7 is provided in the inset.

The first method used illustrates the detection of *E. coli* O157:H7 through the immobilization of antibodies on titania films functionalized with APTES and GA. In the first step (Figure 2a) titania thin films were functionalized with APTES; in particular the spectra of the films before and after the functionalization process were reported. The peaks due to APTES (inset spectrum) have been attributed to N–H stretching at 3300 cm^−1^, N–CH_2_ stretching around 2800 cm^−1^, NH_2_ scissoring and N–H bending at 1615 cm^−1^, aliphatic C–N stretching at 1020–1220 cm^−1^, NH_2_ wagging and twisting at 850–750 cm^−1^ and N–H wagging at 715 cm^−1^.

The second step was based on the reaction between APTES and GA, which was used to crosslink the APTES with antibodies due to the formation of an imine. Here, the terminal amino groups of APTES were changed to aldehydic groups that, in the following step, were covalently coupled with the amino groups of the antibody. The APTES–GA linking is shown in Figure 2b, in which the bands due to the formation of imines in the area between 1900 and 1600 cm^−1^, and the bands related to the stretching of C–N, C–O, C–C groups in the range 1500–1200 cm^−1^ are visible. The GA spectrum is included as a reference in the inset.

**Figure 3:**
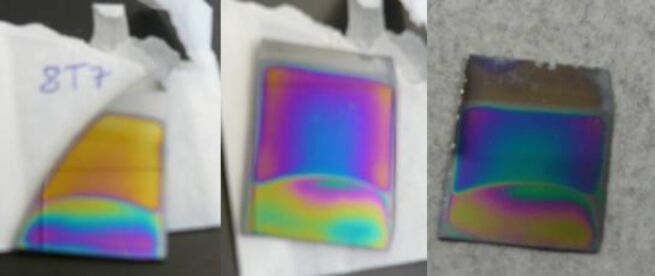
Titania films before functionalization (yellow), after APTES treatment (pink) and after the linking with GA (blue).

**Figure 4:**
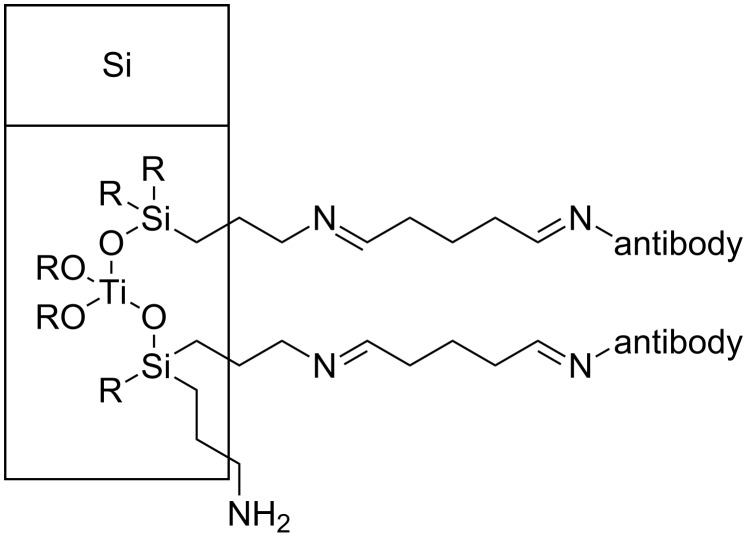
Structure of the chemical linking, TiO_2_–APTES–GA–antibody.

The functionalization process was also visible on the film surface, as reported in Figure 3, due to the change in the colour of films. To complete the sensor fabrication, antibodies against *E. coli* O157:H7 were linked to the substrate as reported in Figure 2c and Figure 4. For the final detection of *E. coli* O157:H7 this chip was immersed in a PBS buffer with *E. coli* O157:H7 (10^8^ CFU/mL) for 30 min, washed and analysed.

The reported spectrum (Figure 2d) shows similar peaks for the film with the pathogen (dotted line) in comparison to films without the pathogen (solid line); however, new peaks appeared in particular in the regions 1300–2000 cm^−1^ (protein peaks of the bacterium), 3700–4000 cm^−1^ and 1200–800 cm^−1^ (signals of nucleic acids of the bacterium), which unfortunately in this region overlapped with the spectrum of the Ab and of the functionalized titania. The peaks in the 1630–1697 cm^−1^ region are due to amide I bands of the proteins in the cell and to their secondary structure. In the region 1402–1457 cm^−1^, bands due to carbohydrates, glycoproteins, lipids and their characteristic C–O–H in-plane bending peaks and C(CH_3_)_2_ symmetric stretching were present. Finally, in the range 900–1100 cm^−1^, peaks due to the DNA/RNA backbone and phosphate groups of nucleic acids due to the symmetric and asymmetric stretching of P=O and P–O–C groups were visible. The spectrum of *E. coli* deposited on Si is provided as a reference in the inset.

### Detection of *E. coli* with other functionalization methods

**Figure 5:**
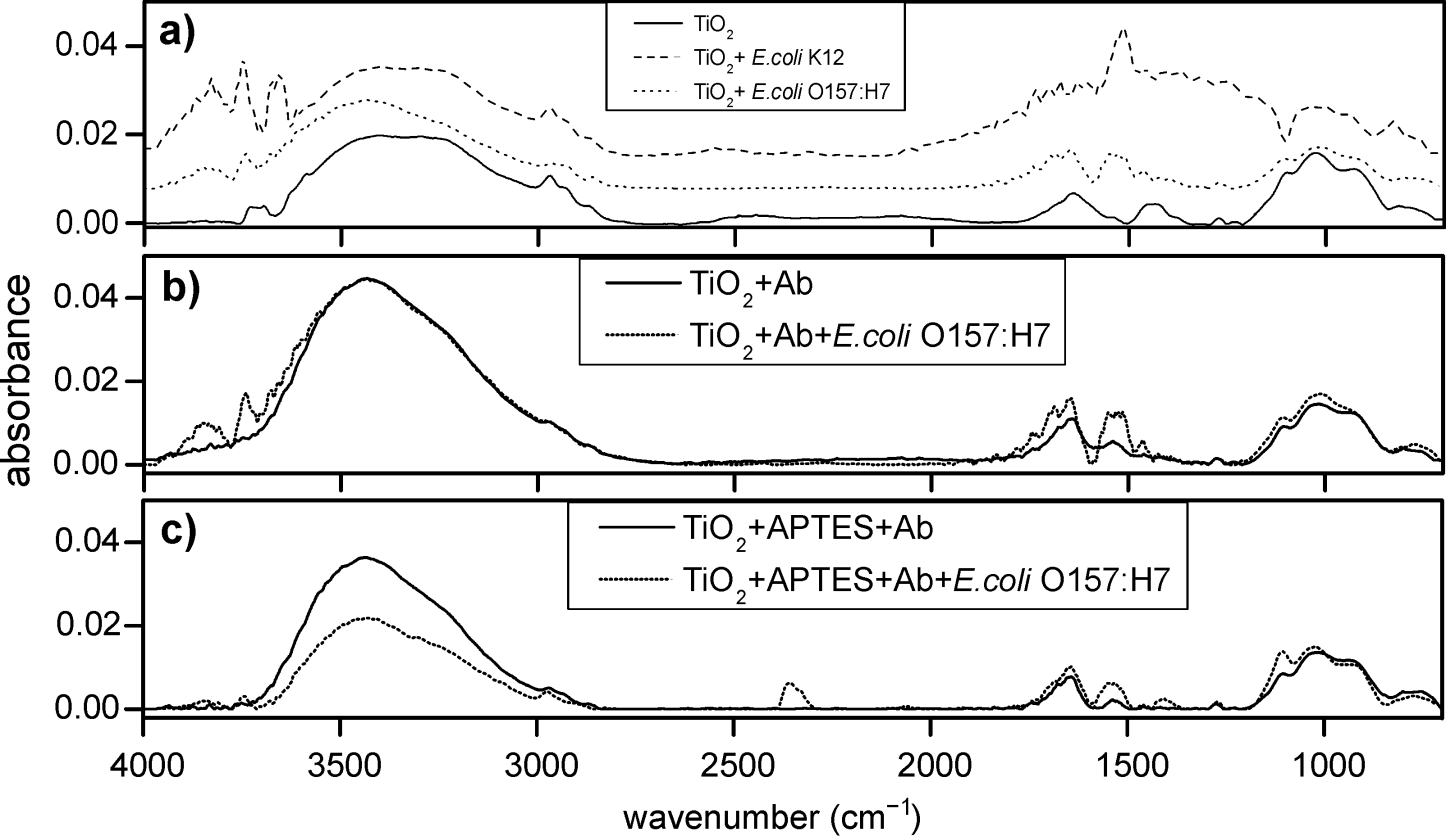
(a) FTIR spectrum of mesoporous titania films before (solid line) and after the immobilization of pathogens *E. coli* O157:H7 (dotted line) and *E. coli* K12 (dashed line). (b) FTIR spectrum of mesoporous titania films functionalized with anti-*E. coli* O157:H7-antibody (solid line) and after the immobilization of the pathogen (dotted line). (c) FTIR spectrum of titania films with APTES and antibody (solid line) and after the immobilization of *E. coli* (dotted line).

To understand the best method for pathogen capture, the direct absorption of the pathogens (*E. coli* O157:H7 and K12) on a titania thin film (Figure 5a), on films with only the specific antibody for *E. coli* O157:H7, and on films with APTES and Ab (Figure 5c) were tested. Figure 5a shows the FTIR spectra of different strains of *E. coli* (O157:H7 and K12) adsorbed onto the film surface with electrostatic interactions, illustrating that the substrate cannot discriminate between different strains and the different spectra of the pathogens are clearly visible. In contrast, in films with specific antibodies, only the binding of *E. coli* O157:H7 was visible and is reported in Figure 5b and 5c, although in films with APTES the presence of pathogens is poor.

Comparing the different immobilization techniques of detection (Figure 2 and Figure 5), the best result was obtained with the method that provided the covalent binding of the Ab on the film surface with APTES and GA (Figure 2d, dotted line), as expected. The other methods allowed the immobilization of bacteria, although not selectively, or with a low sensitivity of the device. In fact only the device shown in Figure 2 is selective and does not allow the linking of other subspecies of *E. coli* (*E. coli* K12), while on titania films (Figure 5a) it was possible to entrap pathogens with electrostatic interactions not differentiating between pathogens.

### Determination of the detection limits of *E. coli* O157:H7

**Figure 6:**
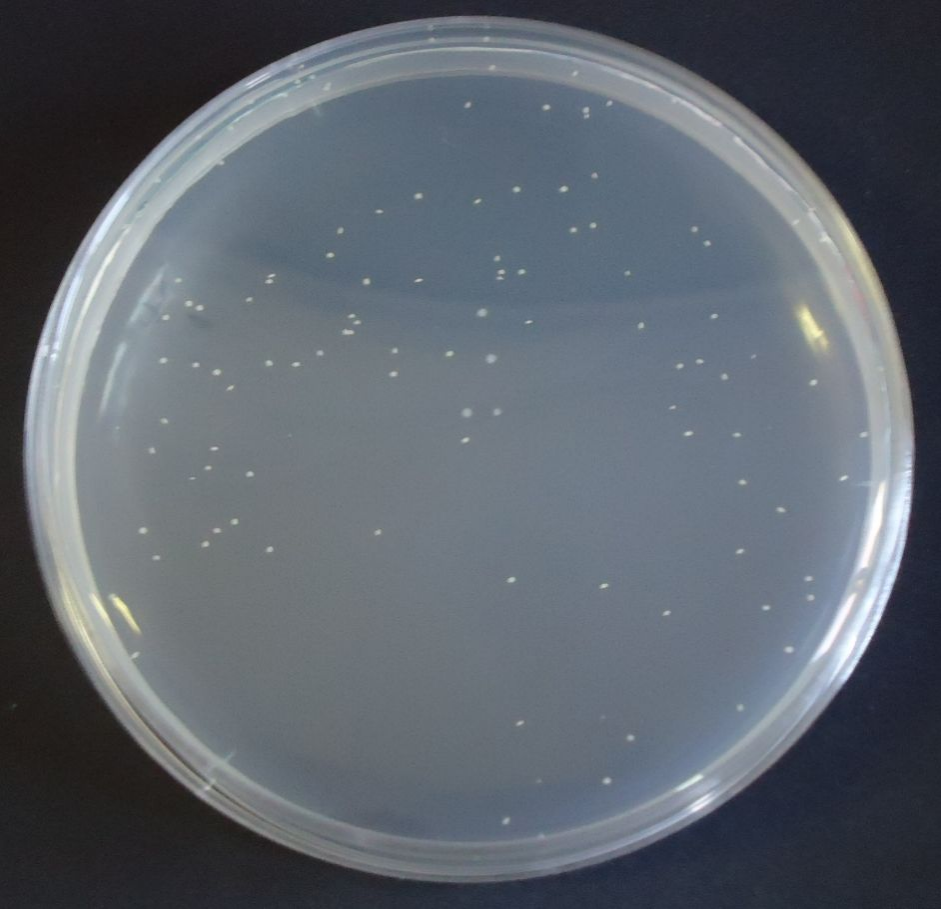
Colony counting method on a Petri plate with PCA and *E. coli* O157:H7 at a dilution of 10^−6^.

**Figure 7:**
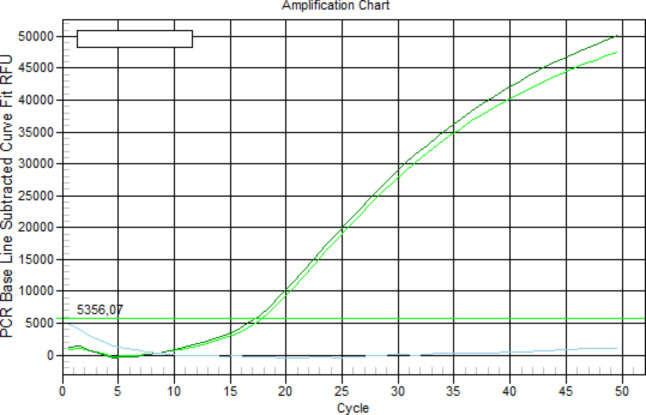
RT–PCR of DNA extracted from the nutrient broth. The blue line is the blank, the light green curve is the reference sample and the green curve is the DNA analysed.

**Figure 8:**
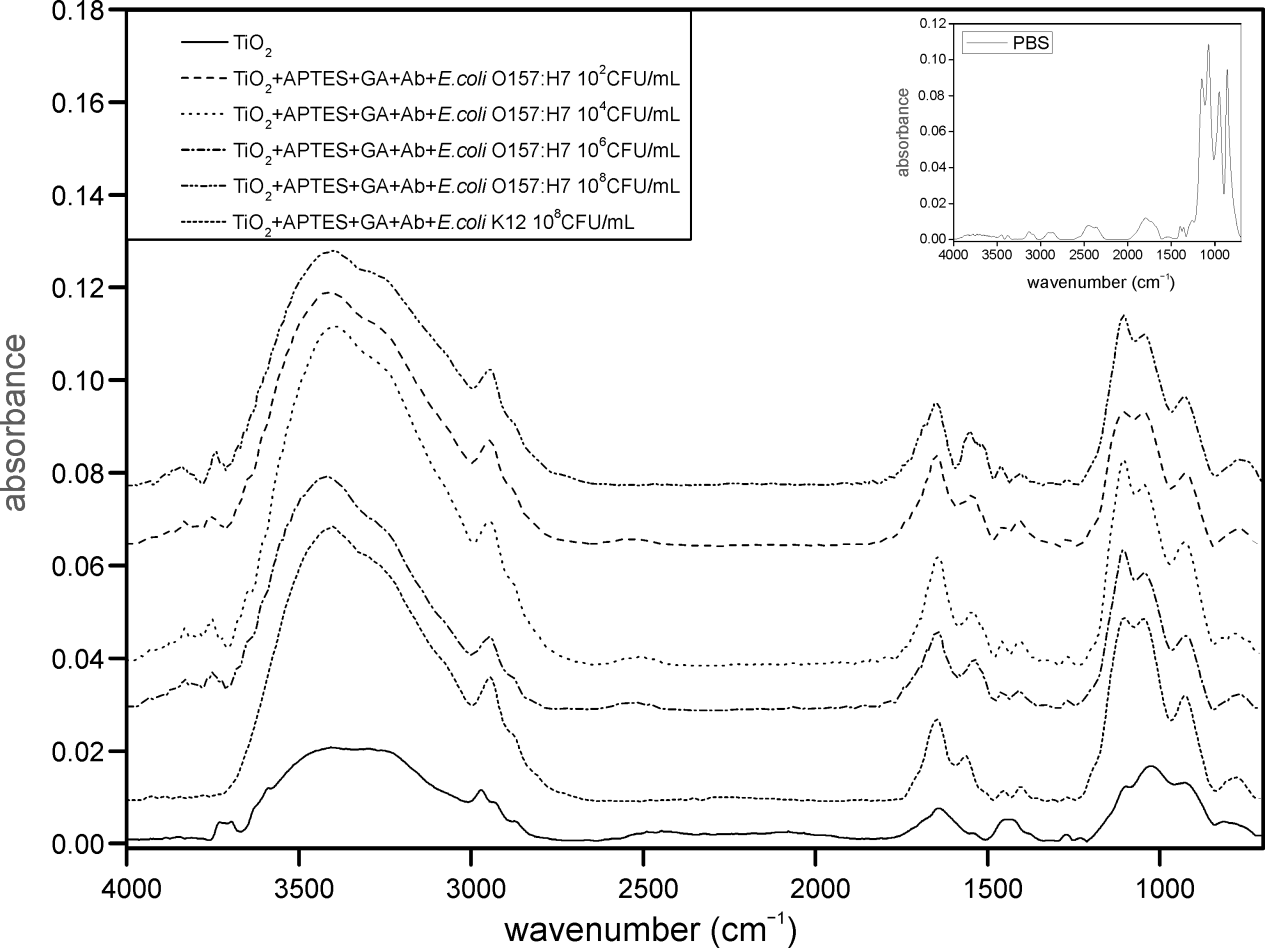
FTIR spectra of titania films alone (solid line) and functionalized with APTES–GA–anti-*E. coli* O157:H7-Ab after exposure to different concentrations of *E. coli* O157:H7 (10^8^–10^2^ CFU/mL) and *E. coli* K12 (10^8^ CFU/mL) in order from the top to the bottom. The negative control of PBS is reported in the inset.

**Figure 9:**
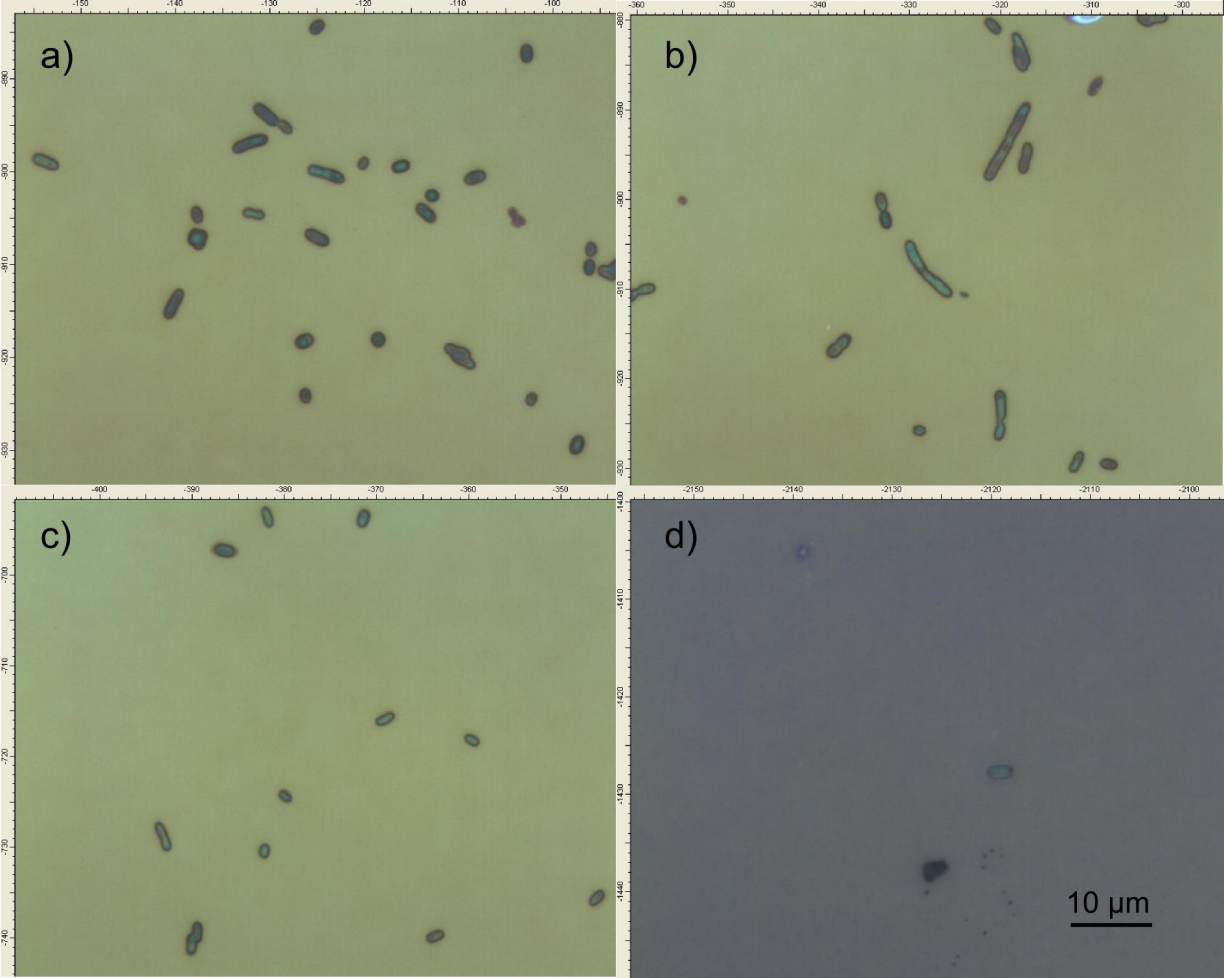
Colony micrographs of *E. coli* O157:H7 immobilized on mesoporous titania films functionalized with APTES–GA–anti-*E. coli* O157:H7-Ab, after exposure to different concentrations of *E. coli* O157:H7. (a) 10^6^ CFU/mL; (b) 10^4^ CFU/mL; (c) 10^2^ CFU/mL; (d) 10 CFU/mL.

Tests to establish the detection limit of the device were carried out using serial dilutions of *E. coli* ranging in concentration from 1 × 10^8^ CFU/mL to 10 CFU/mL; the dilutions were validated with the standard colony counting method (Figure 6) and DNA analysis was achieved by RT–PCR (Figure 7). These experiments were evaluated on mesoporous titania films functionalized with APTES–GA–anti-*E. coli* O157:H7-antibody, and a limit of detection of 1 × 10^2^ CFU/mL (Figure 8) was achieved for *E. coli* O157:H7. A test with *E. coli* K12 was also carried out, but the spectrum of the functionalized chip did not show any peak due to this strain, because of the selectivity of the antibody, illustrating the specificity of the binding. Finally, colony micrographs of functionalized films after the immobilization of *E. coli* O157:H7 at different concentrations were collected with an optical microscope (Figure 9).

## Conclusion

The detection of *E. coli* O157:H7 by using an FTIR platform, through the linking of specific antibodies to mesoporous titania thin films was demonstrated. Cross-linking tethers were used to immobilize antibodies effectively through a chemical method. *E. coli* O157:H7 was identified and classified with standard methods and according to its infrared signature. Nanomaterials used for the immobilization and detection of pathogens, enabled the possible capture of *E. coli* in less than 30 min. The benefit of this approach is specificity due to the antibodies and the characteristic fingerprint of the pathogens. In this way we demonstrated, as with the new biosensor, through a FTIR measurement and a short time for the analysis, that it is possible to discriminate in a selective manner whether a water sample is contaminated with *E. coli* O157:H7. This proposed device can also be adapted for in-field analysis if treated titania films, designed for specific pathogens, can be integrated with portable instruments for the direct assessment of pathogenic contaminants in food systems.
